# Highly selective catalytic reduction of NO via SO_2_/H_2_O-tolerant spinel catalysts at low temperature

**DOI:** 10.1007/s11356-016-7061-y

**Published:** 2016-06-15

**Authors:** Xuanxuan Cai, Wei Sun, Chaochao Xu, Limei Cao, Ji Yang

**Affiliations:** School of Resources and Environmental Engineering, State Environmental Protection Key Laboratory of Environmental Risk Assessment and Control on Chemical Process, East China University of Science and Technology, Shanghai, 200237 China

**Keywords:** SCR, NO_*X*_, Hydrogen, Flue gas, Gas treatment, SO_2_/H_2_O-tolerant

## Abstract

Selective catalytic reduction of NO_*X*_ by hydrogen (H_2_-SCR) in the presence of oxygen has been investigated over the NiCo_2_O_4_ and Pd-doped NiCo_2_O_4_ catalysts under varying conditions. The catalysts were prepared by a sol-gel method in the presence of oxygen within 50–350 °C and were characterized using XRD, BET, EDS, XPS, Raman, H_2_-TPR, and NH_3_-TPD analysis. The results demonstrated that the doped Pd could improve the catalyst reducibility and change the surface acidity and redox properties, resulting in a higher catalytic performance. The performance of NiCo_1.95_Pd_0.05_O_4_ was consistently better than that of NiCo_2_O_4_ within the 150–350 °C range at a gas hourly space velocity (GHSV) of 4800 mL g^−1^ h^−1^, with a feed stream containing 1070 ppm NO, 10,700 ppm H_2_, 2 % O_2_, and N_2_ as balance gas. The effects of GHSV, NO/H_2_ ratios, and O_2_ feed concentration on the NO conversion over the NiCo_2_O_4_ and NiCo_1.95_Pd_0.05_O_4_ catalysts were also investigated. The two samples similarly showed that an increase in GHSV from 4800 to 9600 mL h^−1^ g^−1^, the NO/H_2_ ratio from 1:10 to 1:1, and the O_2_ content from 0 to 6 % would result in a decrease in NO conversion. In addition, 2 %, 5 %, and 8 % H_2_O into the feed gas had a slightly negative influence on SCR activity over the two catalysts. The effect of SO_2_ on the SCR activity indicated that the NiCo_1.95_Pd_0.05_O_4_ possesses better SO_2_ tolerance than NiCo_2_O_4_ catalyst does.

**Graphical abstract:**
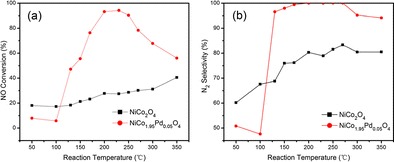
The NiCo_1.95_Pd_0.05_O_4_ catalyst achieved over 90 % NO conversion with N_2_ selectivity of 100 % in the 200∼250 °C range than the maximum 40.5 % NO conversion over NiCo_2_O_4_ with N_2_ selectivity of approximately 80 % in 350 °C.

The NiCo_1.95_Pd_0.05_O_4_ catalyst achieved over 90 % NO conversion with N_2_ selectivity of 100 % in the 200∼250 °C range than the maximum 40.5 % NO conversion over NiCo_2_O_4_ with N_2_ selectivity of approximately 80 % in 350 °C.

## Introduction

Nitrogen oxides (NO_*X*_), including NO, NO_2_, N_2_O, N_2_O_3_, N_2_O_4_, and N_2_O_5_, are mainly derived from fossil fuel combustion (Costa and Efstathiou 2007b). NO_*X*_ are the major air pollutants that are greatly hazardous to human health and the environment (Xiaoling et al. 2012), causing negative effects such as photochemical smog, acid rain, ozone depletion, ground-level ozone, and greenhouse effects (Qi et al. 2006). A number of techniques have been developed to reduce the emission of NO_*X*_. Among them, the selective catalytic reduction of NO_*X*_ by ammonia (NH_3_-SCR) is a well-known and widely industrialized NO_*X*_ control technology for stationary sources such as power plants and nitric acid plants (Li et al. 2010b). Generally, V_2_O_5_-WO_3_ (MO_3_)/TiO_2_ is employed as NH_3_-SCR catalysts (Grossale et al. 2008). However, many problems are encountered in the use of NH_3_-SCR technology, namely catalyst deterioration, NH_3_ slip (emissions of unreacted toxic ammonia), ash odor, air heater fouling, and a high running cost (Olympiou and Efstathiou 2011).

H_2_-SCR has many advantages; for instance, hydrogen as a reductant does not induce any second pollutants and has high activity to reduce NO_*X*_ efficiently at the lowest possible temperature (Kim and Hong 2010; Machida et al. 2001). Especially for industrial sites where H_2_ is readily available, H_2_-SCR is regarded as a possible alternative for NH_3_-SCR. The catalyst is the most central technology in any H_2_-SCR process, and its performance directly affects the removal of nitrogen oxides (Koebel et al. 2001; Weirong et al. 2012). Currently, the catalysts of H_2_-SCR primarily contain supported noble metal oxides (Jun Yub et al. 2003; Yang and Jung 2009), among which the Pt-based and Pd-based catalysts have been revealed to possess good catalytic activity at relatively low temperatures (Chiarello et al. 2007; Costa and Efstathiou 2007a; Qing et al. 2010; Schott et al. 2009). For example, Costa and Efstathiou (2007a) had reported that the Pt/MgO-CeO_2_ catalyst exhibited a maximum of 95 % NO conversion and 78∼92 % N_2_ selectivity within the 100–400 °C range. Higher than 90 % NO_*X*_ conversion within 170–300 °C was presented in the Pd/SiO_2_ catalyst (Qing et al. 2010). Chiarello et al. (2007) investigated the catalytic reduction of NO_*X*_ by H_2_ over Pd-based catalysts with a support consisting of LaCoO_3_. The 0.5 wt% Pd/LaCoO_3_ catalyst exhibited a maximum of approximately 100 % NO conversion and over 78 % N_2_ selectivity at 150 °C.

Nevertheless, noble metals are rare and expensive and they are sensitive to sulfur poisoning. These factors limit their large-scale applications. Therefore, new highly efficient catalysts need to be searched to replace Pt-based and Pd-based catalysts for H_2_-SCR for NO_*X*_. Due to their low price, ready synthesis, and good redox property, transition metal oxides have been widely used as catalysts in various reactions (Auxilia et al. 2014; Chiu et al. 2015). Among them, spinel-type oxides have been widely studied because of their unique structure characteristics, and these oxide catalysts can simultaneously reduce NO_*X*_ and soot at comparatively low temperatures (possibly within the range typical of diesel exhaust 150–380 °C) (Fino et al. 2008). Chen et al. (2009) had reported that the CuCoOx/TiO_2_ catalyst exhibited a maximum of 98.9 % NO conversion at 200 °C. Du et al. (2014) had reported that Co_3_O_4_ showed high performance on the removal of low-concentration (10 ppm) NO at room temperature.

Nickel cobaltite (NiCo_2_O_4_) is a mixed-metal oxide spinel that possesses interesting magnetic properties, rich redox chemistry, good electronic conductivity, and high electrochemical activity (Kim et al. 2000; Rui et al. 2013). In the recent research, NiCo_2_O_4_ is usually explored as an electrode material (Chi et al. 2006; Xu et al. 2014) and has rare reports about the catalytic reduction performance in the SCR reaction. Wang et al. (2015b) had reported that NiCo_2_O_4_ possessed the highest catalytic activity within 50–400 °C, with NO_*X*_ conversion of more than 70 % at 150 °C and N_2_ selectivity of more than 90 % at 100–400 °C. Therefore, we choose nickel cobaltite as a model catalyst.

Generally, doped noble metal can improve the catalytic activity and reduce the operating temperature. Palladium is less expensive and more abundant than platinum (Gaspar and Dieguez 2000), and Pd as an active component shows high activity for H_2_-SCR and is highly active for H_2_ activation (Li et al. 2012). The exceptional performance is due to the high dispersion of Pd in the catalyst that forms the Pd-NO intermediates, which adsorb more NO on the catalyst surface and are reduce to N_2_ by hydrogen with high N_2_ selectivity (Li et al. 2008). Rodríguez and Saruhan (2010) reported that the highly active centers could be formed by the interaction sites between Pd and supports and high NO_*X*_ conversion and N_2_ selectivities could be achieved by the synergistic effects of palladium and perovskites. Moreover, the synergistic effects efficiently enhance H_2_ temperature-programmed reduction (H_2_-TPR). Besides, Xu et al. (2015) had researched that doped Pd could make NiFe_1.95_Pd_0.05_O_4_ increase acidity, reducibility, and catalytic activity.

NO was selected as a model nitrogen oxide in the simulated gas (Wang et al. 2009), because nitrogen monoxide accounts for 95–99 % of all nitrogen oxide emissions in flue gas (Fritz and Pitchon 1997). And the Pd-doped nickel cobaltite catalyst was successfully prepared through a sol-gel auto-ignition method and exhibited good catalytic performance for the selective catalytic reduction of NO by H_2_ in the presence of oxygen at a low temperature. And we have considered the effects of the gas hourly space velocity (GHSV), NO/H_2_ ratios, and O_2_ feed concentration on the SCR activity. For practical consideration, we also investigated the durability of the catalyst and its tolerance to SO_2_ and H_2_O, respectively.

## Materials and methods

### Catalyst preparation

The catalyst used in this research was prepared via a sol-gel method using inorganic salts. All of the chemicals were of analytical grade and used without further purification. The compounds were weighed by analytical balance with the molar ratio of citric acid/Ni(NO_3_)_3_·6H_2_O/Co(NO_3_)_3_·6H_2_O/PdCl_2_ = 3:1:1.95:0.05. Then, these compounds were dissolved in 50 mL deionized water with a concentration of 0.1 mol nitrate precursor (Jauhar et al. 2013). The pH of the mixture solution was additionally adjusted to 5–6 by slowly adding the ammonia solution, then the resulting solution was mixed together under stirring at room temperature for 3 h, and the sol was heated at 80 °C to form a wet gel; afterwards, the gel was dried at 130 °C. After drying, the obtained material was ground into fine powder. In order to obtain crystallized NiCo_1.95_Pd_0.05_O_4_, the powder was calcinated in Muffle furnace at 400 °C for 4 h. Spinel NiCo_2_O_4_ was prepared in similar way with the only difference that merely the molar ratio of citric acid/Ni(NO_3_)_3_·6H_2_O/Co(NO_3_)_3_·6H_2_O/PdCl_2_ was 3:1:2.

According to the drying and roasting process, the particles were easy to reunite and affect the catalyst activity, so about 5 mL polyethylene glycol (PEG) 400 was added to ensure high specific surface area and uniform particle size (Fan and Huang 2011).

### Catalyst characterization

The as-prepared products were characterized by powder X-ray diffraction (XRD) using a Rigaku D/Max 2550 diffractometer (Japan) with Cu Kα radiation (*λ* = 1.54056 Å) operating at 40 kV and 100 mA. The elemental composition of the samples was characterized by energy-dispersive spectrometer (EDS) using a Falion 60s spectrometer. The Brunauer–Emmett–Teller (BET) surface area, average particle size, and pore size of the catalysts were measured with a nitrogen adsorption instrument (Micromeritics, TriStar II 3020) using N_2_ gas as an adsorbent at the temperature of liquid nitrogen. Prior to BET analysis, the samples were degassed at 300 °C for 3 h.

The X-ray photoelectron spectroscopy (XPS) analysis was conducted in a Quantum 2000 Scanning ESCA Microprobe (Physical Electronics). The instrument uses a focused monochromatic Al Kα X-ray (1486.7 eV) source operated at a 100 W and 100-μm-diameter beam. The binding energy scale was calibrated using the carbon C1s at 284.6 eV for known standards. The de-convolution of XPS peak was performed with the CasaXPS program. The Raman spectroscopy experiments were carried out on an Iuvia microscope instrument (Iuvia Reflerx) with a wavelength of 514.5 nm.

H_2_-TPR and temperature-programmed desorption of ammonia (NH_3_-TPD) were performed on AutoChem II 2920 equipped with a thermal conductivity detector (TCD) detector. Prior to the H_2_-TPR or NH_3_-TPD analysis, 50 mg of the oven-dried sample was pretreated in a He stream at 300 °C for 60 min to remove the adsorbed H_2_O and other gases followed by cooling to room temperature. After that, the H_2_-TPR analysis was performed using a 10 % H_2_/Ar mixture at a flow rate of 40 mL/min with a heating rate of 10 °C/min to 800 °C, while the NH_3_-TPD analysis was carried out by 10 % NH_3_/He mixture with a total flow rate of 40 mL/min at 50 °C for 60 min. After NH_3_ adsorption, the sample was purged by He (40 mL/min) for another 60 min. The desorption profile was recorded using a TCD by heating the sample to 600 °C at 10 °C/min under a flow of He (50 mL/min).

### Catalyst activity testing

The selective catalytic reduction of NO by hydrogen was carried out in a fixed-bed flow micro-reactor, the catalyst bed temperature was controlled by thermocouple which was interpolated into the fixed-bed reactor, and reactor was heated through an Al-518/518P-type artificial intelligence temperature controller. A sample weighed 1 g, and the reactant gas composites consisted of 1071 ppm NO; 1071–10,710 ppm H_2_ (the concentration was based on the ratio of NO to H_2_); 0–6 % O_2_; 2 %, 5 %, and 8 % H_2_O (when used); 100, 300, and 500 ppm SO_2_ (when used); and the balance N_2_. These gases were fed from compressed cylinders provided by Jia Jie Specialty Gases (Shanghai, China) and adjusted with Brooks thermal mass flow controllers. The catalyst was fixed by silica pellets and quartz wool and placed in the constant temperature zone of the tubular reactor. The total flow of the inlet gas and the gas hourly space velocity were based on the change of gas conditions. The gas effluent stream from the reactor was analyzed by a Chemiluminescent NO–NO_2_–NO_*X*_ Analyzer (Thermo Scientific, model 42i), and the nitric oxide conversion was indicated using the following equation:

1 $$ {X}_{\mathrm{NO}}\left(\%\right)=\frac{{\left[\mathrm{NO}\right]}_{\mathrm{inlet}}-{\left[\mathrm{NO}\right]}_{\mathrm{outlet}}}{{\left[\mathrm{NO}\right]}_{\mathrm{inlet}}}\times 100\% $$

Among them, *X*_NO_ represents the NO conversion and [NO]_inlet_ and [NO]_outlet_ show the inlet and outlet concentrations of NO in the gas mixture at steady state, respectively.

The Thermo Scientific NO–NO_2_–NO_*X*_ Analyzer revealed that the NO_*X*_ was the sum of NO, NO_2_, and less low-state nitrogen oxides, and the ${X}_{{\mathrm{NO}}_X}$ represented the total NO_*X*_ conversion rate.

2 $$ {X}_{{\mathrm{NO}}_X}\left(\%\right)=\frac{{\left[{\mathrm{NO}}_X\right]}_{\mathrm{inlet}}-{\left[{\mathrm{NO}}_X\right]}_{\mathrm{outlet}}}{{\left[{\mathrm{NO}}_X\right]}_{\mathrm{inlet}}}\times 100\% $$

where the [NO_*X*_]_inlet_ and [NO_*X*_]_outlet_ show the inlet and outlet concentrations of NO in the gas mixture at steady state, respectively. The N_2_ selectivity was calculated as follows:

3 $$ {S}_{{\mathrm{N}}_2}\left(\%\right)=\frac{X_{{\mathrm{N}\mathrm{O}}_X}\cdot {\left[\mathrm{NO}\right]}_{\mathrm{inlet}}}{X_{\mathrm{N}\mathrm{O}}\cdot {\left[\mathrm{NO}\right]}_{\mathrm{inlet}}}\times 100\% $$

In this equation, the *X*_NO_·[NO]_inlet_ expresses the amount of nitric oxide in the transformation, while the ${X}_{{\mathrm{NO}}_X}\cdot {\left[\mathrm{NO}\right]}_{\mathrm{inlet}}$ expresses the amount of nitrogen from the transformation of nitric oxide.

## Results and discussion

### Catalyst characterization

#### Structural and textural properties

**Fig. 1:**
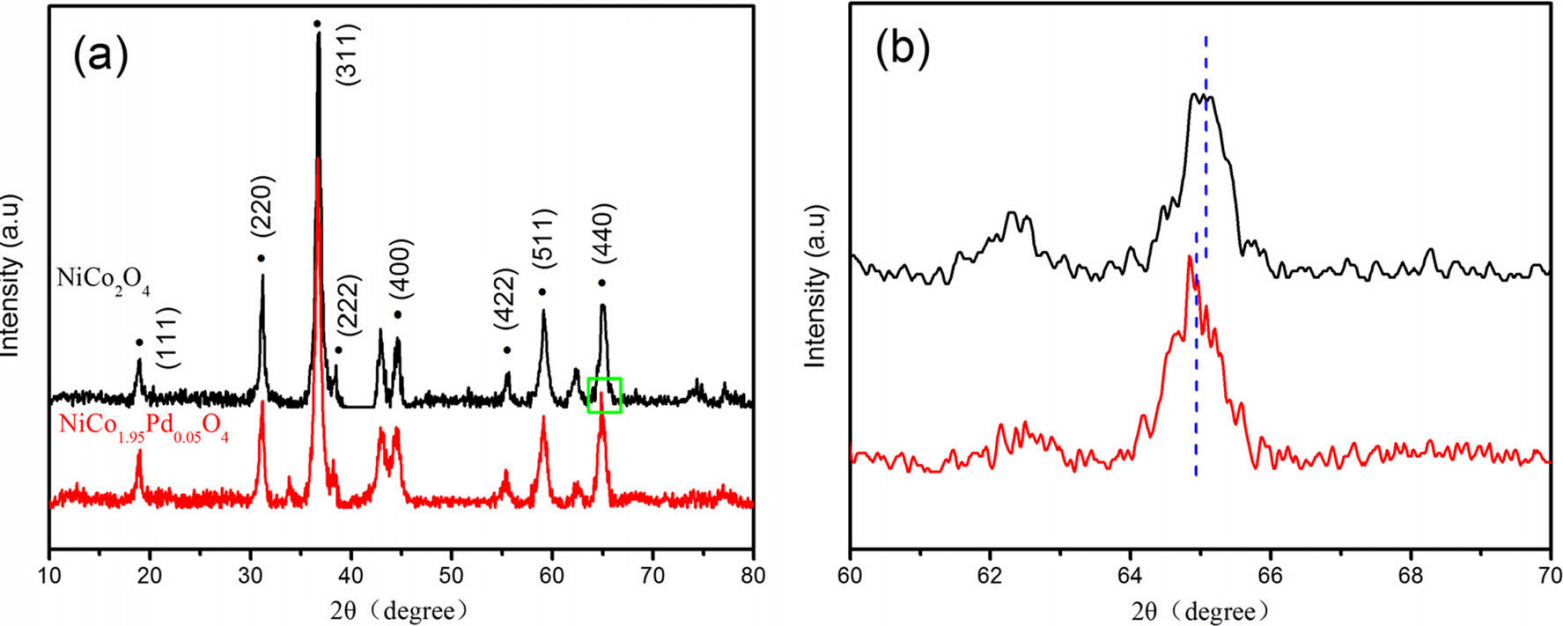
**a** XRD patterns of NiCo_2_O_4_ and NiCo_1.95_Pd_0.05_O_4_ used in this study. **b** XRD peak positions of (440) crystal planes

The XRD patterns of the samples used in this study are shown in Fig. 1a. The major peaks of the samples at ca. 2*θ* = 18.9°, 31.1°, 36.7°, 38.4°, 44.6°, 55.4°, 59.1°, and 65.1° could be indexed to (111), (220), (311), (222), (400), (422), (511), and (440) crystal planes of spinel NiCo_2_O_4_, respectively. It could be seen that the samples were a spinel cubic structure which were in good agreement with the standard pattern (JCPDS No. 73-1702) (Xu et al. 2014). While the XRD pattern of NiCo_1.95_Pd_0.05_O_4_ was similar to that of NiCo_2_O_4_, the diffraction angle theta of (440) crystal planes slightly shifted from 65.1° to 64.9°, which is shown in Fig. 1b. These results indicated that the heavier and larger Pd^2+^ ions substituted the Co^2+^ ions in the structure of the nickel cobaltite spinel, resulting in a small angle deviation of XRD peaks (Kavas et al. 2009). And the Pd doping did not cause a significant change in the crystallinity of the samples, possibly due to the palladium that replaced Co without distorting the spinel structure; therefore, the NiCo_1.95_Pd_0.05_O_4_ sample also maintained the spinel cubic structure.

The average crystalline size of NiCo_2_O_4_ and NiCo_1.95_Pd_0.05_O_4_ was estimated to be 32.2 and 26.7 nm using Scherrer’s formula based on the (422) peak, respectively (Lou et al. 2008). Scherrer’s formula is as follows:

4 $$ D=\frac{K\lambda }{\beta \cos \theta } $$

Among which, *D* is crystalline size, *K* is constant, *λ* is X-ray wavelength (0.154056 nm), *β* is peak half-high width, and *θ* is the diffraction angle theta. Due to nickel cobaltite’s spinel cubic structure, *K* should be changed to 0.943 and a half-high width should be converted into a radian system, [(*β* / 180) × 3.14].

#### BET and EDS analysis

The BET surface areas, average particle size, and pore size of the studied NiCo_2_O_4_ and NiCo_1.95_Pd_0.05_O_4_ were summarized in Table 1. The surface areas of the NiCo_2_O_4_ and NiCo_1.95_Pd_0.05_O_4_ samples were 14.9956 and 12.6566 m^2^ g^−1^, respectively, while the average particle sizes were 14.0774 and 15.4802 nm, respectively, which had the reverse order compared to the surface area values. Generally, the larger the particle size, the smaller the BET area. The elemental composition of the samples was investigated using an EDS, as shown in Table 1. The Co/Ni atomic ratio (we chose Ni for comparison due to the stoichiometric amount being 1 in all of the studied samples) for NiCo_2_O_4_ was 2.03, which was consistent with the stoichiometric ratio. Due to the palladium doping, the Co/Ni atomic ratio of NiCo_1.95_Pd_0.05_O_4_ was 1.92, deviating slightly from the theoretical ratio of 1.95. The Pd/Ni atomic ratio was 0.049, which was consistent with the theoretical value of 0.05.

**Table 1:** Physical and chemical properties of the studied catalysts

Catalysts	*S*_BET_ (m^2^ g^−1^)	*D*_particle_ (nm)	*D*_pore_ (nm)	Elemental composition (at.%)				
				Ni	Co	Pd	Pd/Ni	Co/Ni
NiCo_2_O_4_	14.9956	400.1164	14.0774	18.78	38.26	0.00	0.000	2.037
NiCo_1.95_Pd_0.05_O_4_	12.6566	474.0594	15.4802	20.29	38.97	1.00	0.0493	1.921

#### H_2_-TPR and NH_3_-TPD analysis

**Fig. 2:**
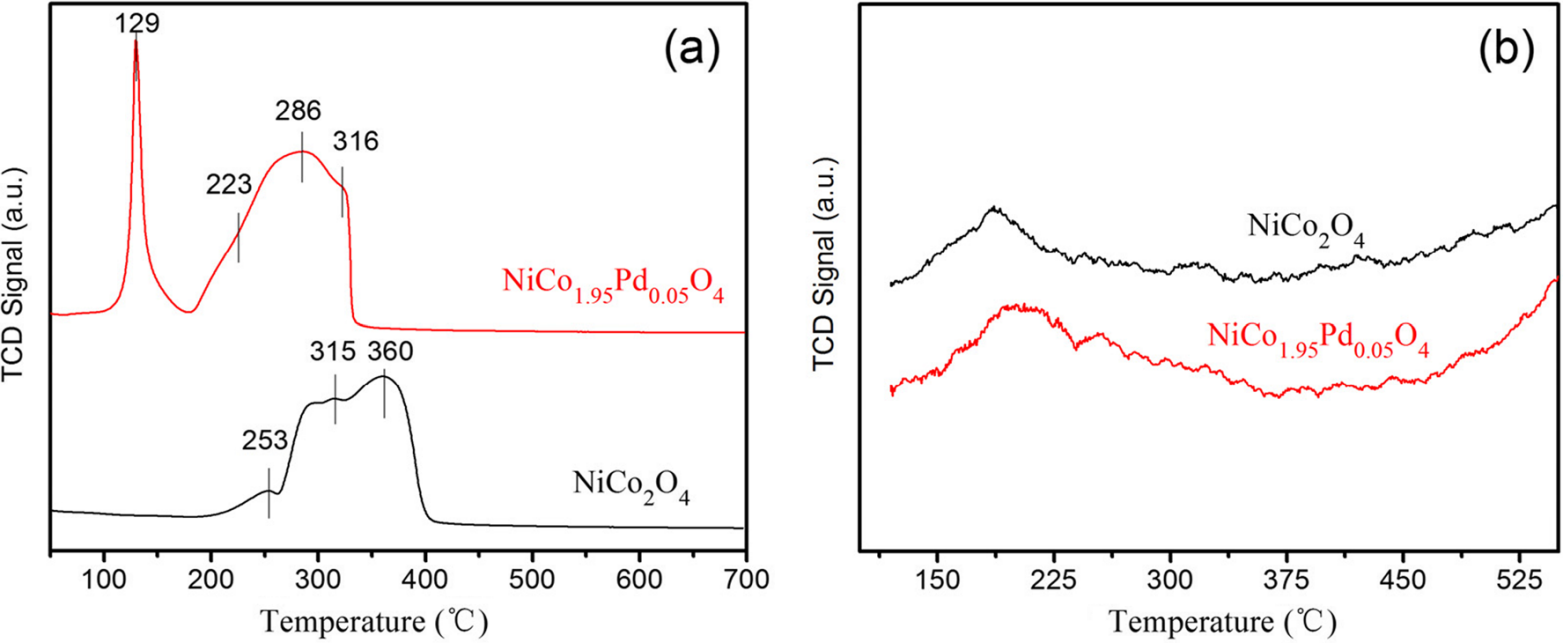
H_2_-TPR and NH_3_-TPD profiles of NiCo_2_O_4_ and NiCo_1.95_Pd_0.05_O_4_ used in this study. **a** H_2_-TPR. **b** NH_3_-TPD

In order to investigate the reducibility and acidic properties of the NiCo_2_O_4_ and NiCo_1.95_Pd_0.05_O_4_, the as-prepared samples were characterized by H_2_ temperature-programmed reduction (H_2_-TPR) and temperature-programmed desorption of ammonia (NH_3_-TPD) analysis, respectively. As shown in Fig. 2a, there were three distinct reduction peaks in NiCo_2_O_4_, and the peaks appeared at 253 and 360 °C, corresponding to the reduction steps of Co^3+^ to Co^2+^ and Co^2+^ to Co^0^, respectively (Gou et al. 2013; Lim et al. 2015), while the peak appeared at 315 °C, which was attributed to the reduction of Ni^2+^ to Ni^0^. However, in comparison to NiCo_2_O_4_, the H_2_-TPR profile of the NiCo_1.95_Pd_0.05_O_4_ catalyst showed one slight peak and one strong broad peak. The slight peak at 129 °C was assigned to the reduction of Pd^2+^ to Pd^0^ (Giraudon et al. 2007; Ling et al. 2015), which was observed in pure Ni-Co spinel. And the strong broad peak within the range of 200–350 °C was formed by three reduction peaks fully overlapped, which corresponded to the reductions of Co^3+^ to Co^2+^ at 223 °C, Ni^2+^ to Ni^0^ at 286 °C, and Co^2+^ to Co^0^ at 316 °C. Compared to NiCo_2_O_4_, the reduction peaks of NiCo_1.95_Pd_0.05_O_4_ shifted to low temperature, which indicated that the H_2_ consumption was raised, enhancing the redox properties of catalysts. In addition, the TPR peak areas were an indicator to determine the reducibility of catalyst; the greater the peak area, the stronger the reducibility. The peak areas of H_2_-TPR were provided in Table 2. It could be seen that the Pd-containing nickel cobaltate exhibited relatively higher TPR areas than NiCo_2_O_4_, indicating that the palladium doping could improve the catalyst reducibility.

**Table 2:** The peak areas of H_2_-TPR and NH_3_-TPD

Catalysts	H_2_-TPR								Total	NH_3_-TPD	
	Peak 1		Peak 2		Peak 3		Peak 4			Peak	
	*T* (°C)	Area	*T* (°C)	Area	*T* (°C)	Area	*T* (°C)	Area		*T* (°C)	Area
NiCo_2_O_4_	253	3.1764	315	11.5537	360	17.7736	0	0.0000	32.5037	186	0.2000
NiCo_1.95_Pd_0.05_O_4_	129	11.1585	223	4.3780	286	26.4388	316	3.5521	45.5274	206	0.3189

The unit of the peak area was defined as 1. The dosage of the samples was the same in the measurement of H_2_-TPR and NH_3_-TPD

The acidic properties of the as-prepared samples were shown in Fig. 2b. The NH_3_ adsorbed on the acid sites and then desorbed at different temperature regions, which was determined by the strength of acid sites and the amount of acid, respectively. The NH_3_ desorption below 400 °C corresponded to the weak- and medium-strength acid sites (Imran et al. 2013). Nevertheless, the TPD profile of NiCo_2_O_4_ showed a broad NH_3_ desorption peak from 100 to 350 °C, which was assigned to the NH_3_ desorbed by weak- and medium-strength acid sites. Moreover, the acid amount could be determined by the TPD peak areas and the size of peak area corresponded to the amount of acid. Table 2 showed the NH_3_-TPD peak areas. Compared to NiCo_2_O_4_, NiCo_1.95_Pd_0.05_O_4_ slightly increased the peak areas from 100 to 350 °C, indicating that NiCo_1.95_Pd_0.05_O_4_ showed a relatively more acidic amount than NiCo_2_O_4_.

#### Raman analysis

**Fig. 3:**
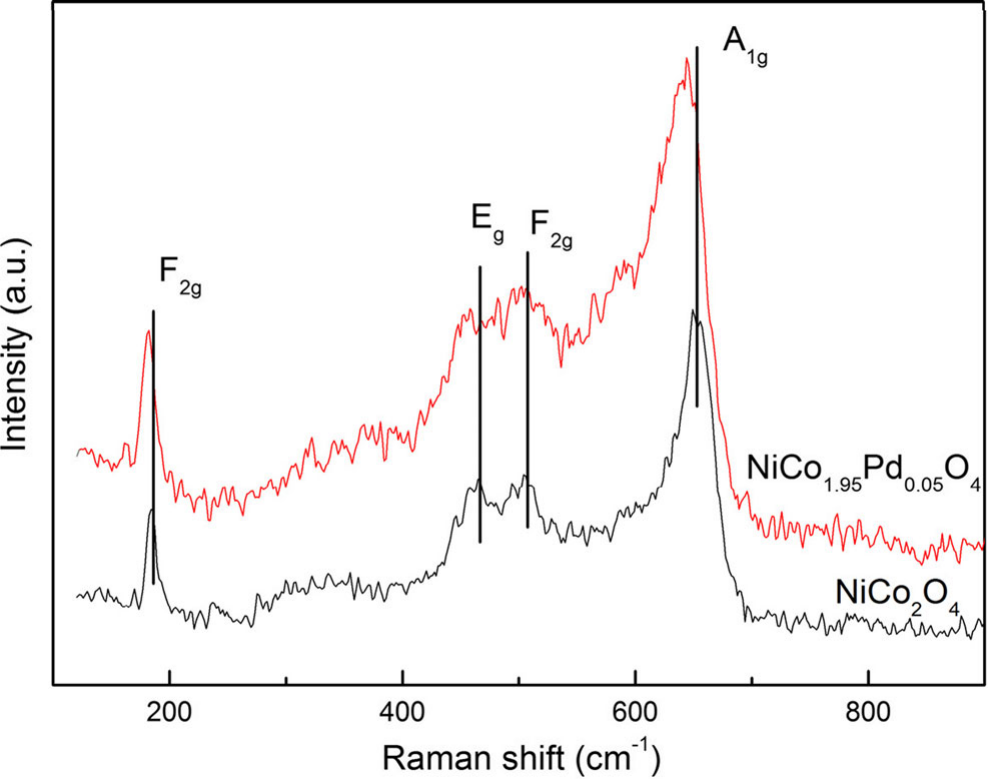
Raman profiles of NiCo_2_O_4_ and NiCo_1.95_Pd_0.05_O_4_ used in this study

NiCo_2_O_4_ is an inverse spinel with tetragonal (A site) positions occupied by mostly Co^3+^ and octahedral (B site) positions occupied by nearly equal concentrations of Ni^2+^ and Co^3+^ (Iliev et al. 2013). In order to further understand the composition and structural features of the NiCo_2_O_4_ and NiCo_1.95_Pd_0.05_O_4_, the samples were characterized with Raman spectroscopy. The typical Raman spectrum was shown in Fig. 3; the peaks of NiCo_2_O_4_ at 183, 465, 509, and 651 cm^−1^ corresponded to F_2g_, E_1g_, F_2g_, and A_1g_ models of NiCo_2_O_4_, respectively, and the Co–O and Ni–O stretching vibrations could be detected in the Raman spectrum. These results were well consistent with previously reported literatures (Babu et al. 2014; Li et al. 2015; Liu et al. 2013). Moreover, the peaks at 181, 418, 501, and 644 cm^−1^ corresponded to F_2g_, E_1g_, F_2g_, and A_1g_ models of NiCo_1.95_Pd_0.05_O_4_, respectively. Compared with that of NiCo_2_O_4_, the Raman spectra of the Pd-substituted nickel cobaltate (NiCo_1.95_Pd_0.05_O_4_) exhibited a shift toward lower wave number, which was the result of the heavier and larger Pd^2+^ ions substituting the Co^2+^ ions in the structure of the nickel cobaltite spinel.

#### XPS analysis

**Fig. 4:**
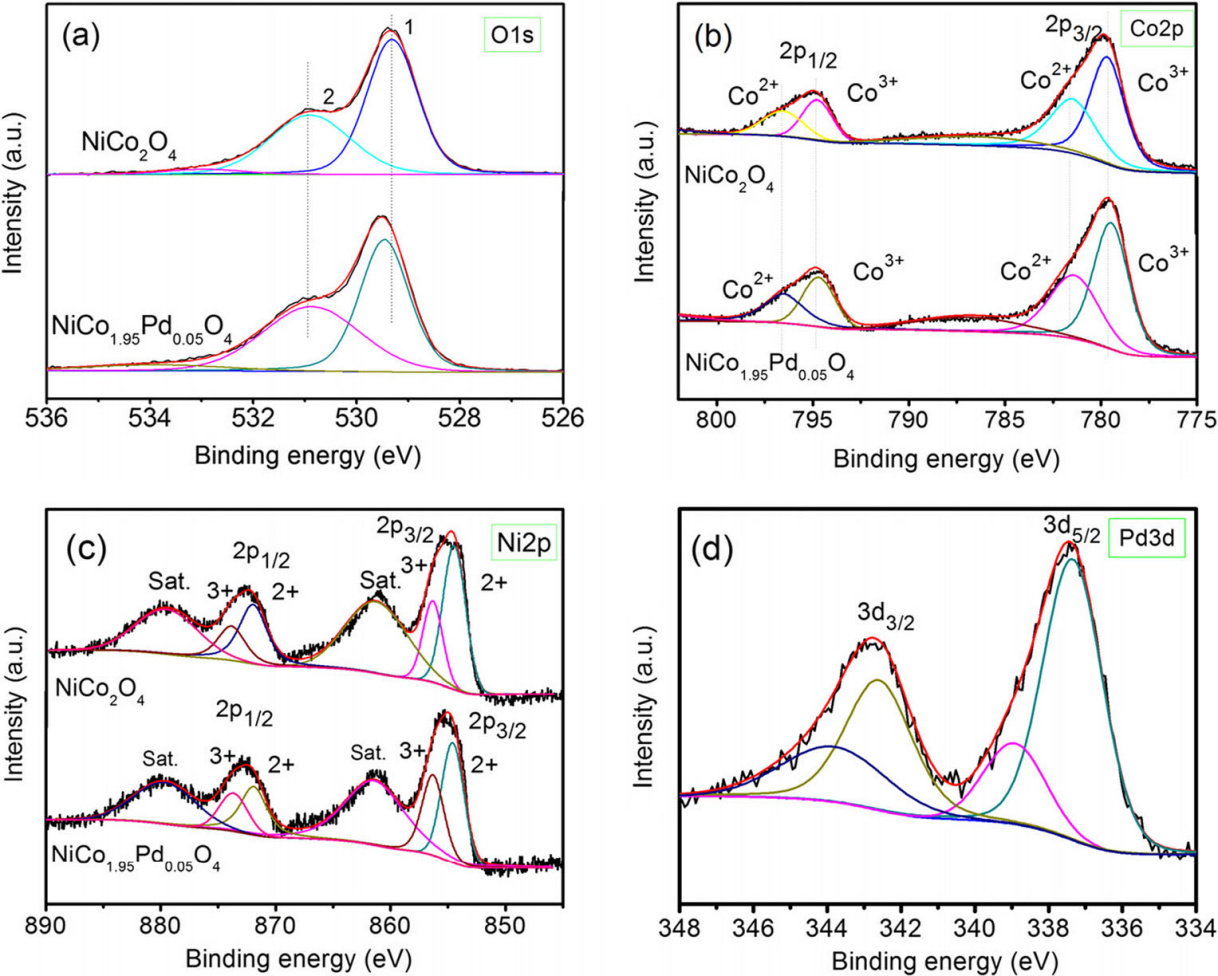
XPS spectra of NiCo_2_O_4_ and NiCo_1.95_Pd_0.05_O_4_ used in this study. **a** O1s. **b** Co2p. **c** Ni2p. **d** Pd3d

In order to investigate the chemical bonding states and compositions of surface elements of as-synthesized NiCo_2_O_4_ and NiCo_1.95_Pd_0.05_O_4_, the samples were studied by X-ray photoelectron spectroscopy (XPS), and the results were shown in Fig. 4a–d. The Ni, Co, O, and Pd elements were detected for the prepared samples.

The high-resolution spectrum for the O1s region in Fig. 4a showed two peaks at binding energies of around 529.1 and 530.8 eV, respectively, which had been denoted as O1 (529.1 eV) and O2 (530.8 eV). The O1 at the low energy of 529.1 eV was typical of the O atoms in the O–Co/Ni bonding. And the O_2_ at 530.8 eV was a characteristic of the as-prepared NiCo_2_O_4_ samples, which corresponded to a number of defect sites with low-oxygen coordination in the material with small particle size (Kim et al. 2000; Liu et al. 2013; Rui et al. 2013). The Co2p binding energies and peak shape were similar for the two preparations (Fig. 4b) and yield binding energies of 779.8 and 794.7 eV for the 2p3/2 and 2p1/2 transitions, respectively (Kim et al. 2000). XPS spectra of Co2p3/2 in the two preparations showed two main peaks of binding energies at 779.4 and 781.4 eV, which were assigned to the surface Co^3+^ and Co^2+^ species, respectively (Wang et al. 2015b). And this indicated that there were only a few Co^2+^ species in the octahedral sites and most of the low-spin Co^3+^ species occupied the octahedral sites (Babu et al. 2014; Kim et al. 2000).

The Ni2p spectra given in Fig. 4c were fitted considering two spin-orbit doublets as a characteristic of Ni^2+^ and Ni^3+^ and two shake-up satellites. The fitting peaks at the binding energy of 854.4 and 871.6 eV were indexed to Ni^2+^, while the fitting peaks at the binding energy of 856.43 and 873.8 eV were ascribed to Ni^3+^, respectively (Li et al. 2015, 2010a; Yu et al. 2016). The Pd3d spectra, as presented in Fig. 4d, showed two peaks at the binding energy of 337.1 eV for 3d5/2 and 342.5 eV for 3d3/2, respectively. The Pd5/2 binding energy was closed to the value of 336.9 which is a characteristic of Pd^2+^, indicating that the Pd doped in the as-prepared NiCo_1.95_Pd_0.05_O_4_ sample (Giraudon et al. 2007; Hu et al. 2011; Ling et al. 2015).

These results exhibited that the surface of the as-synthesized NiCo_2_O_4_ that contained Ni^2+^, Ni^3+^, Co^2+^, and Co^3+^ (Moni et al. 2014), while the NiCo_1.95_Pd_0.05_O_4_ also contained Pd^2+^ except the common elements.

### Catalyst performance

#### SCR activity of the NiCo_2_O_4_ and NiCo_1.95_Pd_0.05_O_4_ catalysts

**Fig. 5:**
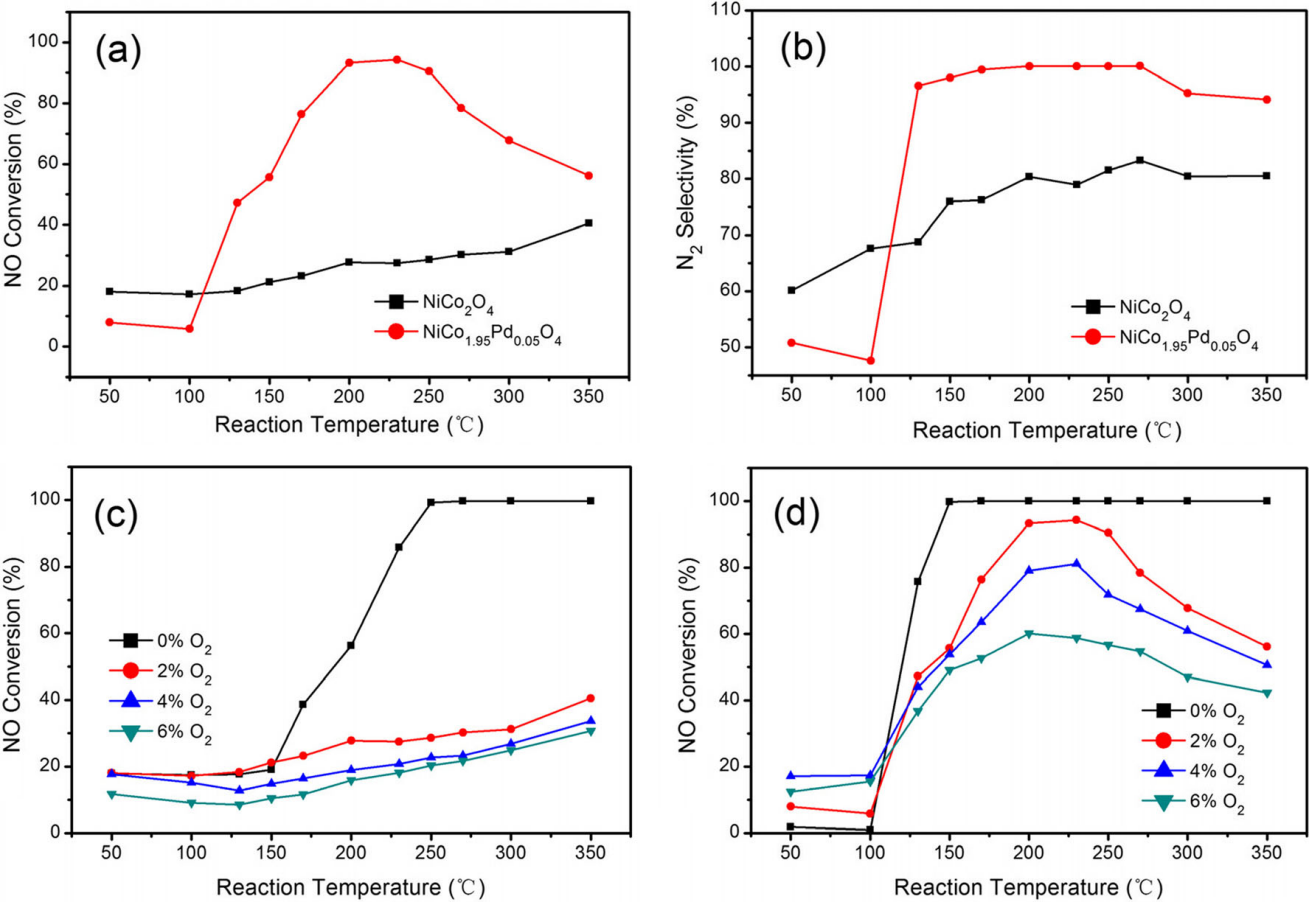
**a** The effect of the reaction temperature on NO conversion for H_2_-SCR over the NiCo_2_O_4_ and NiCo_1.95_Pd_0.05_O_4_ catalysts in the presence of 2 % O_2_. **b** N_2_ selectivity over the NiCo_2_O_4_ and NiCo_1.95_Pd_0.05_O_4_ catalysts in the presence of 2 % O_2_. **c**, **d** The effect of oxygen concentration (0∼6 %) on NO conversion for H_2_-SCR over the NiCo_2_O_4_ and NiCo_1.95_Pd_0.05_O_4_ catalysts. Reaction condition: [NO] = 1070 ppm; [H_2_] = 10,700 ppm; [O_2_] = 0 %, 2 % (**a**, **b**), 4 %, and 6 %; [N_2_] = balance; catalyst mass = 1 g; GHSV = 4800 mL g^−1^ h^−1^; and *T* = 50–350 °C. **c** NiCo_2_O_4_. **d** NiCo_1.95_Pd_0.05_O_4_

Figure 5 shows the results of the NO conversion and N_2_ selectivity over the NiCo_2_O_4_ and NiCo_1.95_Pd_0.05_O_4_ in the temperature range of 50–350 °C with a GHSV of 4800 mL g^−1^ h^−1^ in the presence of 2 % O_2_. As shown in Fig. 5a, as the reaction temperature increased, the NO conversion firstly increased then decreased over the NiCo_1.95_Pd_0.05_O_4_ catalyst. While the SCR activity over the NiCo_2_O_4_ catalyst was always increasing until it reached the maximum NO conversion. The highest NO conversion of the NiCo_2_O_4_ catalyst was approximately 40.05 % at 350 °C; yet, the N_2_ selectivity over the NiCo_2_O_4_ catalyst was only 60∼80 %, which is illustrated in Fig. 5b. The NO conversion over the NiCo_1.95_Pd_0.05_O_4_ catalyst was higher than 90 % at a suitable temperature of 200–250 °C with the maximum of 94.65 % at 230 °C. And the N_2_ selectivity was higher than 90 % in the whole 130–350 °C range, with the maximum closed to 100 % at the 200–270 °C range.

Moreover, a previous study showed that the content of O_2_ was a crucial parameter for the H_2_-SCR reaction (Yang et al. 2011; Yuan et al. 2013). The combustion of fuel was not sufficient in the old burners, which resulted in the high oxygen content of 6∼12 %. However, the new type of circulating fluidized bed burns the fuel more efficiently, resulting in the low oxygen content of 4∼6 % (Huilin et al. 2000). Hence, in our study, the highest oxygen content was chosen to be 6 %. In general, oxygen can inhibit NO removal efficiency by oxidizing H_2_ to H_2_O, but it also oxidizes NO into adsorbed nitrite/nitrate on the surface to enhance the reduction reaction by hydrogen in the H_2_-SCR process (Machida et al. 2001; Wei et al. 2012). Hence, the effect of the oxygen concentration ([O_2_] = 0 %, 2 %, 4 %, and 6 %) in the feed gas over the NiCo_1.95_Pd_0.05_O_4_ and NiCo_2_O_4_ catalysts was investigated, which also consisted of 1070 ppm NO/10,700 ppm H_2_/*x* % O_2_/N_2_ at a temperature range of 50–350 °C with a GHSV of 4800 mL g^−1^ h^−1^.

The oxygen content in the flue gas from the circulating fluidized bed boiler is generally 4∼6 % (Huilin et al. 2000). Therefore, experiments were performed to examine the O_2_ tolerance of the prepared catalysts. As illustrated in Fig. 5c, under O_2_-free reaction conditions, a higher removal efficiency of NO was achieved and an efficiency of 100 % over the NiCo_2_O_4_ catalysts was stabilized within a temperature range of 250–350 °C. As the O_2_ content increased from 0 % to 6 %, the deNO_*X*_ performance rapidly decreased and the maximum NO conversion decreased from 100 to 30.77 % at the 350 °C. As shown in Fig. 5d, the high NO conversion of 100 % over NiCo_1.95_Pd_0.05_O_4_ was stabilized within a wider temperature range of 150–350 °C in the absence of oxygen, indicating that the doped Pd improved the catalytic activity and shifted the reaction temperature of the efficiency of 100 % from 250 to 150 °C. When the O_2_ increased to 2 %, the maximum NO conversion over NiCo_1.95_Pd_0.05_O_4_ only changed to 94 % at the 230 °C; as the O_2_ content increased, the SCR still exhibited good activity for NO reduction, with the maximum NO conversion of 81.16 % with 4 % O_2_ at 230 °C and 61 % in the presence of 6 % O_2_ at 230 °C, proving that Pd incorporation not only significantly improves the catalytic activity but also increases the O_2_ tolerance ability (Wei et al. 2012).

To sum up, the NiCo_1.95_Pd_0.05_O_4_ catalyst showed higher catalytic performance as well as greatly reduced the reaction temperature of the maximum removal efficiency. Hence, the NiCo_1.95_Pd_0.05_O_4_ catalyst was suitable for the selective catalytic reduction of NO by H_2_ in the presence of oxygen at a low temperature. Therefore, Pd played a very important role in the nickel cobaltite catalyst in the H_2_-SCR reaction. In addition, palladium did not destroy the spinel structure of NiCo_2_O_4_ when it doped in nickel cobaltite, which was consistent with the XRD results. And from the H_2_-TPR and NH_3_-TPD activity tests, the NiCo_1.95_Pd_0.05_O_4_ exhibited relatively high TPR areas, reduction level, and slightly larger acidity than NiCo_2_O_4_. All these factors resulted in the better catalytic performance of NiCo_1.95_Pd_0.05_O_4_.

#### Effect of GHSV and NO/H_2_ ratio

**Fig. 6:**
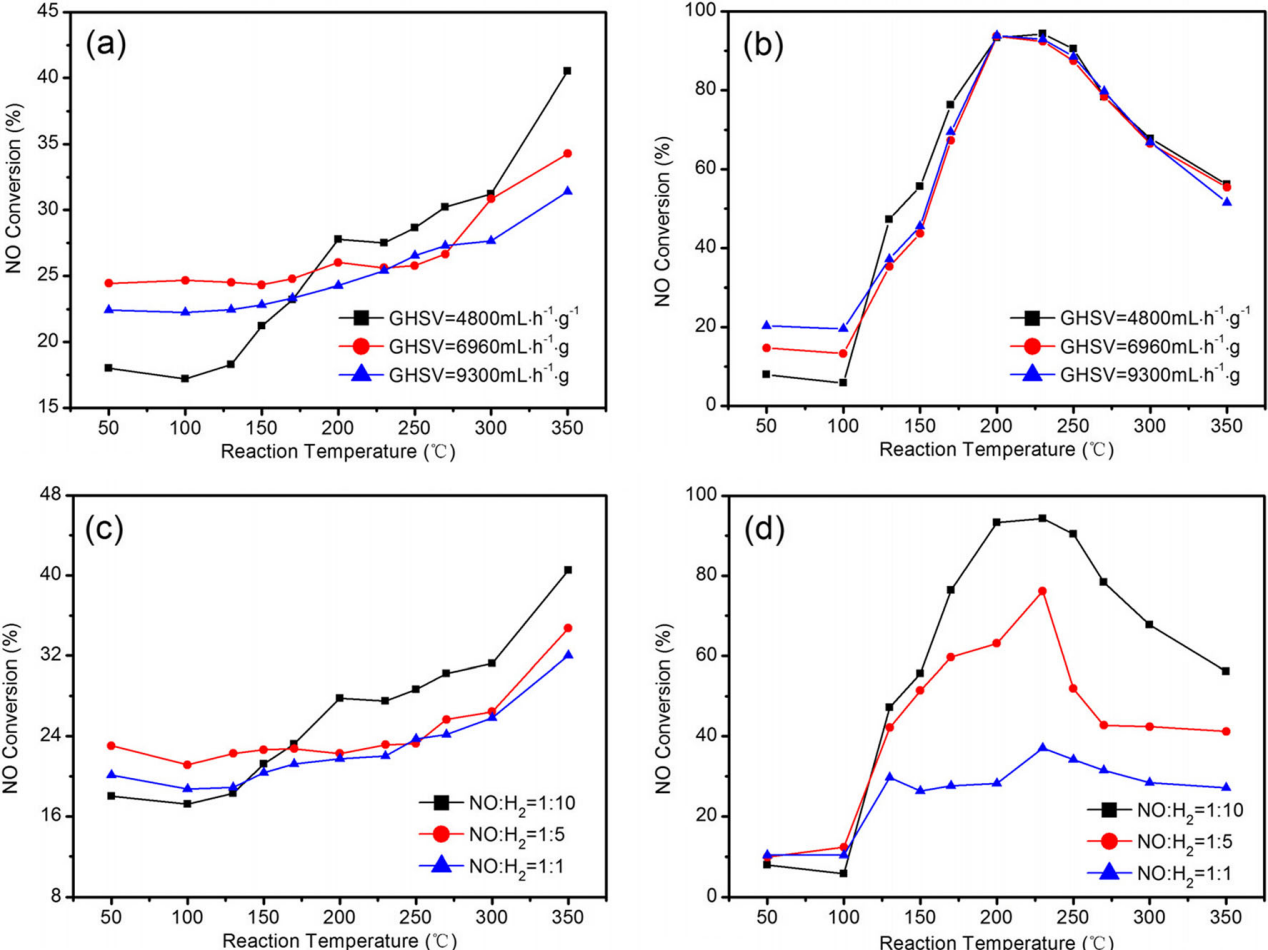
Effect of gas hourly space velocity and NO/H_2_ ratios on NO conversion for H_2_-SCR over the NiCo_1.95_Pd_0.05_O_4_ and NiCo_2_O_4_ catalysts. Reaction condition: [H_2_] = 10,700 ppm (when examined gas hourly space velocity), [NO] = 1070 ppm, [O_2_] = 2 %, [N2] = balance, catalyst mass = 1 g, GHSV = 4800 mL g^−1^ h^−1^ (when NO/H_2_ ratios were examined), and *T* = 50–350 °C. **a** NiCo_2_O_4_. **b** NiCo_1.95_Pd_0.05_O_4_. **c** NiCo_2_O_4_. **d** NiCo_1.95_Pd_0.05_O_4_

In general, GHSV could significantly affect the NO conversion rate at low temperature, and it was believed that it has less effect on the conversion rate at high temperature (Qi et al. 2006). Consequently, the H_2_-SCR activity of the NiCo_2_O_4_ and NiCo_1.95_Pd_0.05_O_4_ catalysts at different GHSVs (from 4800 to 9300 mL h^−1^ g^−1^) and NO and H_2_ feed concentration ratios (NO/H_2_ = 1:10–1:1) were investigated at a temperature range of 50–350 °C in the presence of 2 % O_2_, and the results are shown in Fig. 6.

As shown in Fig. 6a, the NO conversion over the NiCo_2_O_4_ catalyst decreased with the increasing GHSV. When the GHSV was 4800 mL h^−1^ g^−1^, the maximum NO conversion was up to 40.5 % at 350 °C, while when the GHSV increased to 6960 and 9300 mL h^−1^ g^−1^, the maximum conversion decreased to 35 % and 31 %, respectively. However, in contrast with NiCo_2_O_4_, the NO conversion faintly decreased over the NiCo_1.95_Pd_0.05_O_4_ catalyst with the increase of the GHSV from 4800 to 9600 mL h^−1^ g^−1^ at the reaction temperature of 125–350 °C, which was shown in Fig. 6b. When the GHSV was 4800 mL h^−1^ g^−1^, the maximum NO conversion was up to 94 % at 230 °C, and when the GHSV was 9300 mL h^−1^ g^−1^, the maximum conversion was approximately 92 % at 230 °C.

These results demonstrated that the GHSV was a crucial parameter for the H_2_-SCR reaction and the space velocity determined the residence time of the gas in the catalyst. As GHSV increased, the residence time of feed gas decreased. The NiCo_1.95_Pd_0.05_O_4_ catalyst showed high NO conversion with the increasing GHSV, due to the Pd addition, which enhanced the redox properties of the active catalyst component. And this was consistent with H_2_-TPR. Although the increase of GHSV resulted in the decrease of the reaction gas residence time in the catalyst, the activity of the active component was enhanced in the Pd-doped catalyst, resulting in more reduction reaction sites for NO and only a slight decrease of NO conversion. Therefore, the Pd-doped NiCo_1.95_Pd_0.05_O_4_ catalyst showed better catalytic performance than the NiCo_2_O_4_ catalyst at different GHSVs (from 4800 to 9300 mL h^−1^ g^−1^).

The variation trend of NO conversion over the sample catalysts was roughly similar as the increase of reaction temperature at different NO/H_2_ ratios, the NO conversion firstly increased then decreased over the NiCo_1.95_Pd_0.05_O_4_ catalyst with the increasing reaction temperature, but the removal of NO over the NiCo_2_O_4_ catalyst increased as the temperature increased. As shown in Fig. 6c, the NO conversion over the NiCo_2_O_4_ catalyst decreased as the NO and H_2_ feed concentration ratio increased within a temperature range of 170–350 °C in the presence of 2 % O_2_. When the NO/H_2_ ratio was 1:10, the maximum NO conversion was 41 %. When the NO/H_2_ ratio was changed to 1:5 and 1:1, the NO conversion rate was 34 % and 31 %, respectively, exhibiting that higher H_2_ concentration that resulted in higher NO conversion. Nevertheless, compared with the NiCo_2_O_4_ catalyst, the NiCo_1.95_Pd_0.05_O_4_ catalyst showed higher catalytic activity at different NO and H_2_ ratios, which is shown in Fig. 6d. When the NO/H_2_ ratio was 1:10, the maximum NO conversion was more than 95 % at a reaction temperature of 230 °C. Actually, the economic factors must be considered. Industrially, the lower NO/H_2_ ratio was used to obtain a relatively high NO conversion. When the NO/H_2_ ratio was changed to 1:5 and 1:1, the NO conversion rate was 76 % and 37 %, respectively. Therefore, the doped Pd largely improved the catalytic activity and NiCo_1.95_Pd_0.05_O_4_ exhibited higher catalytic performance than NiCo_2_O_4_ in the common reaction condition.

#### Effect of the presence of H_2_O and SO_2_

H_2_O and SO_2_ were usually contained in the industrial flue gases, which could cause a deactivation on SCR catalysts, and the general content of water was 2∼10 %. At the same time, the SCR catalysts are sensitive to sulfur poisoning since sulfur compounds could deposit on the active sites of catalysts and deactivate them irreversibly (Chang et al. 2013; Lee et al. 2013; Yin et al. 2015). Therefore, it was important to investigate the effect of H_2_O and SO_2_ on NO conversion over selected catalysts.

**Fig. 7:**
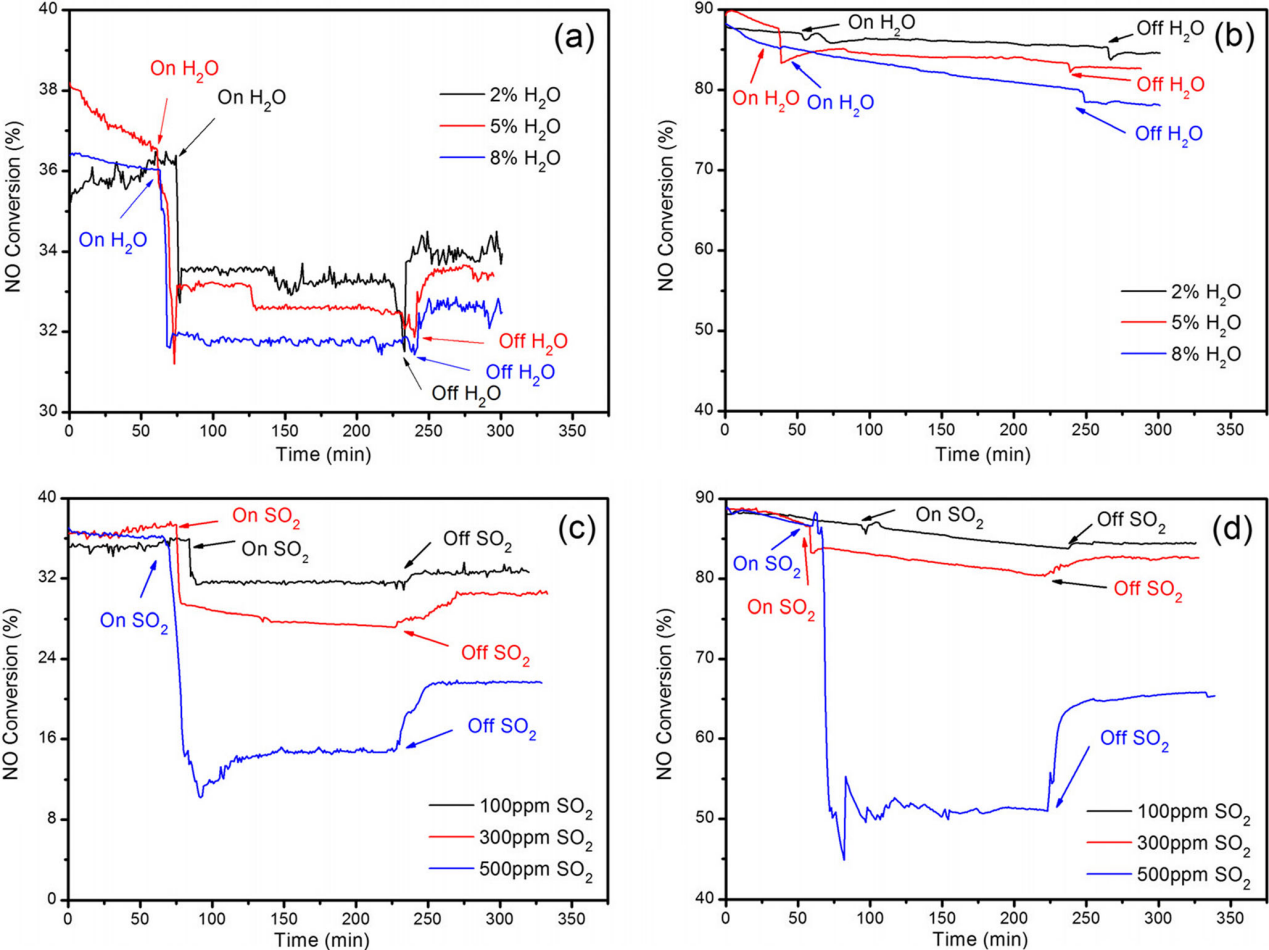
Effect of H_2_O ([H_2_O] = 2 %, 5 %, and 8 %) and SO_2_ ([SO_2_] = 100, 300, and 500 ppm) on NO conversion for H_2_-SCR over the NiCo_2_O_4_ and NiCo_1.95_Pd_0.05_O_4_ catalysts. Reaction condition: [NO] = 930 ppm (950 ppm, when SO_2_ was added), [H_2_] = 9300 ppm (9500 ppm, when SO_2_ was added), [O_2_] = 2 %, [N2] = balance, catalyst mass = 1 g, GHSV = 9300 mL g^−1^ h^−1^, and flow rate = 155 mL/min. **a** NiCo_2_O_4_, *T* = 350 °C, when H_2_O was added. **b** NiCo_1.95_Pd_0.05_O_4_, *T* = 230 °C, when H_2_O was added. **c** NiCo_2_O_4_, *T* = 350 °C, when SO_2_ was added. **d** NiCo_1.95_Pd_0.05_O_4_, *T* = 230 °C, when SO_2_ was added

The effect of H_2_O and SO_2_ on the selective catalytic reduction of nitric oxide with hydrogen over the NiCo_2_O_4_ and NiCo_1.95_Pd_0.05_O_4_ catalyst was demonstrated in Fig. 7. As shown in Fig. 7a, when 2 %, 5 %, and 8 % H_2_O was introduced to feed gas, the NO conversion of NiCo_2_O_4_ decreased rapidly from 36.8 %, 36.5 %, and 36.3 % to 33.2 %, 32.5 %, and 31.8 %, respectively, and then the NO conversion recovered slowly to 34.2 %, 33.4 %, and 32.6 % after the removal of H_2_O, respectively. Compared with the initial value, the decrement of NO conversion was 7.07 %, 8.49 %, and 12.95 %, respectively. It could be seen that the conversion decreased more as the content of H_2_O increased, which was due to the water-irreversible dissociative adsorption on the active sites of the catalyst (Burch and Coleman 1999; Leicht et al. 2012). And the NO conversion recovered a little, which was due to the reversible competitive adsorption by water. Hence, the influence of water on the catalyst was both reversible and irreversible.

However, the behavior of H_2_O poisoning of the NiCo_1.95_Pd_0.05_O_4_ catalyst was quite different. As shown in Fig. 7b, after 2 %, 5 %, and 8 % H_2_O was introduced into the inlet gas, the NO conversion showed a slight decrease approximately from 87.2 % to 86.5 %, 84.5 %, and 80.5 %, respectively, which probably was due to the H_2_O competitive adsorption with NO as well as with H_2_. The water on the catalyst could form the additional surface hydroxyls because of the dissociative adsorption of water (Kijlstra et al. 1996), and the surface hydroxyls would neutralize the acid sites, resulting in the reduction of SCR activity. For the Pd-doped catalyst, the surface acidity was enhanced. The multi-acid neutralized the hydroxyls and decreased the water poisoning on active sites of the catalyst. Therefore, the NiCo_1.95_Pd_0.05_O_4_ catalyst has a better H_2_O tolerance than NiCo_2_O_4_. Furthermore, the influence of surface hydroxyls neutralizing the acid sites was irreversible; when the H_2_O was off, the acidity of acid sites could not recover, and hence, the NO conversion over the NiCo_1.95_Pd_0.05_O_4_ catalyst could not restore to the original level, which was consistent with Fig. 7b.

Moreover, the SO_2_ tolerance and regenerability of NiCo_2_O_4_ and NiCo_1.95_Pd_0.05_O_4_ catalysts were also examined. As shown in Fig. 7c, when the 100 ppm SO_2_ was added to the reactant gas, the NO conversion of NiCo_2_O_4_ at 350 °C decreased rapidly from 35.8 % to 31.6 % in 150 min and only recovered to 32.6 % after the removal of SO_2_, manifesting the inhibition effect of SO_2_ on the SCR activity over NiCo_2_O_4_ (Chang et al. 2013; Yin et al. 2015). When the SO_2_ increased to 300 and 500 ppm, the NO conversion decreased rapidly to lower than 27.5 % and 14.5 % and only recovered to 30.6 % and 21.6 % after the removal of SO_2_, respectively. The decrease of NO conversion was mainly due to the blocking of the active sites by the formation of metal sulfates and/or sulfites (Li et al. 2011). The sulfated species formed on the catalytic center inhibited the SCR activity, which resulted in the decrease of NO conversion. After the supply of SO_2_ was cut off, the NO conversion recovered slowly in a certain amount, which was mainly due to the regeneration of the part of sulfated catalysts by the hydrogen (Wang et al. 2015a).

The SO_2_ poisoning behavior to the NiCo_1.95_Pd_0.05_O_4_ catalyst at 230 °C was quite similar. As shown in Fig. 7d, after 100, 300, and 500 ppm SO_2_ was introduced to the inlet gas, the NO conversion of NiCo_1.95_Pd_0.05_O_4_ decreased steadily from 88.1 % to 83.8 %, 80.5 %, and 51.6 % in 130 min, respectively. The decrement of NO conversion was 4.88 %, 8.63 %, and 41.43 % compared to the initial value; however, the decrement value of NiCo_2_O_4_ was 11.73 %, 23.18 %, and 59.50 %, respectively. This was attributed to the improvement of catalytic activity by enhanced surface acidities and redox properties, which was consistent with NH_3_-TPD and H_2_-TPR. After 100, 300, and 500 ppm SO_2_ was off, the NO conversion of NiCo_1.95_Pd_0.05_O_4_ could efficiently recover to 84.4 %, 82.8 %, and 65.5 %, respectively, indicating that the deactivation was partially reversible.

These results demonstrated that the NiCo_1.95_Pd_0.05_O_4_ catalyst possesses better SO_2_ tolerance than NiCo_2_O_4_ catalyst does.

## Conclusions

In this study, the NiCo_2_O_4_ and NiCo_1.95_Pd_0.05_O_4_ catalysts were investigated for NO selective reduction by hydrogen in the presence of O_2_. The results showed that the NiCo_2_O_4_ catalyst had a certain effect for NO removal, while the Pd-containing NiCo_1.95_Pd_0.05_O_4_ catalyst exhibited better SCR activity with the maximum NO conversion of 94.65 % at 230 °C and the N_2_ selectivity of 100 %. Moreover, the prepared NiCo_1.95_Pd_0.05_O_4_ exhibited higher H_2_O and SO_2_ tolerance than NiCo_2_O_4_.
